# Waiting and seeing: The importance of long‐term system‐wide monitoring of mouse models of disease

**DOI:** 10.1002/hem3.70087

**Published:** 2025-01-31

**Authors:** David G. Kent

**Affiliations:** ^1^ Department of Biology, Centre for Blood Research, York Biomedical Research Institute University of York York UK

Researchers undertaking complex in vivo research ranging from assessing hematopoietic stem cell (HSC) function to complex disease modeling are all too familiar with the pressures to find a phenotype and publish the resulting data as soon as possible. Moreover, the vagaries of the academic system are such that the individual driving the project is also often under pressure to deliver the first story and move on to the next project, making it doubly difficult to delve deeper into the biology of a specific mouse model. Therefore, the field has many exciting mouse models where only the surface has been scratched with respect to the biological function of a particular mutation. Running counter to this is a recent paper in *HemaSphere* ^1^ from the McKinney‐Freeman lab that explores a mouse model of G‐protein‐coupled receptor‐associated sorting proteins (GPRASPs) longitudinally for its wider effects on the hematopoietic system. The paper “GPRASP protein deficiency triggers lymphoproliferative disease by affecting B‐cell differentiation”^1^ is a direct follow‐up on the exciting story detailing GPRASP functional consequences in HSCs that appeared several years ago in *Blood*.^2^ This new study in *HemaSphere* shows the power of undertaking detailed biological assessments of a mouse model beyond the initial findings in the area of research that a specific lab has expertise and is a great example of the power of fully characterizing and monitoring mouse models.

GPRASPs were identified as candidate molecules for altering HSC function due to their expression in long‐term HSCs and evidence in other tissues of influencing and mediating microenvironmental changes. With previous functions highlighted in development and the maintenance of homeostasis, they represented strong targets for functional validation. The McKinney‐Freeman lab took the first steps by individually silencing GPRASP family members in the setting of HSC transplantation to test whether GPRASP loss of function would enhance homing and function of HSCs. They showed increased survival, quiescence, migration, and homing and further went on to detail that GPRASPs were involved in the degradation of CXCR4, which meant that their removal increased the stability of CXCR4, a known regulator of HSC lodgement and homing in the adult bone marrow.^2^ Notably, CXCR4 is the main chemokine receptor for stromal‐derived factor 1 (SDF1), the key driver of the HSC chemotaxis that allows bone marrow homing to occur and is highly expressed on HSCs. That said, CXCR4 is also expressed in a range of other cells, and this is where the story gets interesting in this study.

Many researchers would have stopped the study here and moved on to a new model, but as shown in the recent issue of *HemaSphere*, Morales‐Hernández has now followed up the mouse model and discovered another exciting role for GPRASPs in hematological disease. In this paper, they show that the downregulation of GPRASP1 and GPRASP2 also affects maturing B cells, which require CXCR4. The result of CXCR4 accumulation in this case is retention in the germinal centre and coincident increased activation‐induced cytidine deaminase (AID) expression, which drives somatic hypermutation. In turn, this leads to the striking observation of B cell malignancies in mice that receive GPRASP‐deficient cells. This system now offers the unique opportunity to study initial transforming events that might more accurately reflect the broad mutational spectra of B‐cell malignancies, which do not traditionally have the same level of high‐frequency driver mutations observed in their myeloid counterparts. Modulating GPRASP function alongside genetic profiling of the B cells with increased somatic hypermutation could unlock a range of new mechanistic insights into the origins of B‐cell malignancies that we would otherwise be completely blind to.

Overall, this study underscores the tenacity of the research team in fully characterizing their mouse model and also highlights the critical need to explore the function of broadly expressed molecules such as CXCR4 through common mediators like the GPRASPs. The fundamental process of cellular adherence in a local microenvironment is the probable linkage in this case, but many such “HSC regulators” or immune cell regulators have not been tested in other cell types and could be powerfully utilized to make additional discoveries about the biology of hematological malignancies.

## AUTHOR CONTRIBUTIONS

David G. Kent is the sole contributor to this article.

## CONFLICT OF INTEREST STATEMENT

The author declares no conflict of interest.

## FUNDING

Work in the D. G. K. laboratory is supported by the Bill and Melinda Gates Foundation (INV002189), a Cancer Research UK Programme Foundation Award (DCRPGF\100008), Blood Cancer UK (24041), and the Medical Research Council (MC_PC_21043; MR/Y011945/1).

## Data Availability

Data sharing is not applicable to this article as no data sets were generated or analyzed during the current study.
